# Incidence and risk factors of gastrointestinal neuroendocrine neoplasm metastasis in liver, lung, bone, and brain: A population‐based study

**DOI:** 10.1002/cam4.2567

**Published:** 2019-10-14

**Authors:** Zhibo Zheng, Chuyan Chen, Lingjuan Jiang, Xingtong Zhou, Xiaoyan Dai, Yimin Song, Yongning Li

**Affiliations:** ^1^ Department of International Medical Services Peking Union Medical College Hospital Chinese Academy of Medical Sciences Beijing China; ^2^ Department of Gastroenterology Peking Union Medical College Hospital Chinese Academy of Medical Sciences Beijing China; ^3^ Central Research Laboratory Peking Union Medical College Hospital Chinese Academy of Medical Sciences Beijing China; ^4^ Department of Surgery Peking Union Medical College Hospital Chinese Academy of Medical Sciences Beijing China

**Keywords:** gastrointestinal, metastases, neuroendocrine neoplasms, SEER

## Abstract

**Background:**

Neuroendocrine neoplasm is a rare solid tumor. Metastatic pattern of the gastrointestinal neuroendocrine neoplasm (GI‐NEN) has not been fully explored.

**Methods:**

Data were obtained from the Surveillance, Epidemiology, and End Results (SEER) database (SEER‐9 registry) from 1973 to 2015. Incidence was estimated by Joinpoint regression analyses. Data with additional treatment fields of GI‐NEN were extracted from the SEER‐18 registry from 1 January 2010 to 31 December 2015. A total of 14 685 GI‐NEN patients were included in this study. Statistical analyses were performed with SPSS 25.0, the Intercooled Stata SE 15.0, and GraphPad Prism 7.

**Results:**

Incidence of GI‐NENs increased from 0.51 per 100 000 patients in 1973 to 6.20 per 100 000 patients in 2015. Of them, 2003 patients were stage IV GI‐NEN at the time of diagnosis, including 1459 (72.84%) patients with liver metastasis, 144 (7.19%) lung metastasis, 115 (5.74%) bone metastasis, and 27 (1.35%) brain metastasis. Esophageal NEN had the highest risk of metastasis (52.68%). The median survival for patients with liver, lung, bone, and brain metastasis was 38, 6, 9, and 2 months, respectively. The presence of lung or liver metastasis indicated higher risk of concurrent existence of bone and brain metastasis than those without.

**Conclusion:**

Bone and brain metastasis should be screened in the GI‐NEN patients if they had lung or liver metastasis. Findings of the current study could help clinicians to identify distant metastasis of GI‐NENs as early as possible, and by which, to improve survival rate of GI‐NENs.

## Graphical abstract

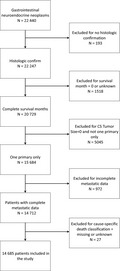

## 1 INTRODUCTION

Neuroendocrine neoplasms (NENs) are solid tumors that originate from neuroendocrine cells. According to the recent population‐based studies, the incidence of gastrointestinal NENs (GI‐NENs) has increased substantially in the past several decades.^1^, ^2^, ^3^, ^4^ The epidemiology, diagnosis, and treatment of NENs have been well‐studied, whereas studies on the metastasis of GI‐NENs remain relatively scarce. Neuroendocrine neoplasms generally grow slowly and silently, rendering patients with a relatively longer survival time. Therefore, metastases have become the main reason causing cancer‐related death of NENs.

Previous studies revealed that 13.17% of GI‐NENs were diagnosed at stage IV once metastasis was noticed.^5^ Liver is the most common metastatic site of GI‐NENs, followed by the lung, bone and brain.^6^, ^7^ Brain metastasis from GI‐NENs is rare, and most of our knowledge on bone or brain metastasis from GI‐NENs come from case reports.^8^ Due to the limitation of sample size, studies describing the epidemiology, risk factors, and survival of metastatic GI‐NENs are limited.

The Surveillance, Epidemiology, and End Results (SEER) Program of the United States National Cancer Institute (NCI) provided a population‐based information on cancer statistics from 1973 until now. Since 2010, data of the liver, lung, bone, and brain metastases of various types of cancer have been collected and available for analysis. The study was aimed to analyze the metastatic spectrum of primary site‐specific metastases of GI‐NENs in SEER program from 2010 to 2015, and to search for possible risk factors of metastasis. Providing statistical evidence for metastatic patterns of GI‐NENs may help physicians to make better decision at pretreatment evaluation stage.

## 2 METHODS

### 2.1 Study population

The SEER program of the NCI collects information on cancer incidence and survival from population‐based cancer registries of several geographic regions in the US. Available data include patient demographics, tumor characteristics (histology, grading, Tumor‐Node‐Metastasis stage), treatment and patients' vital status. The SEER database started to release cancer metastatic information related to the liver, lung, bone and brain from 2010.

SEER*Stat 8.3.5 software was used to extract information from the database.

**Figure 1:**
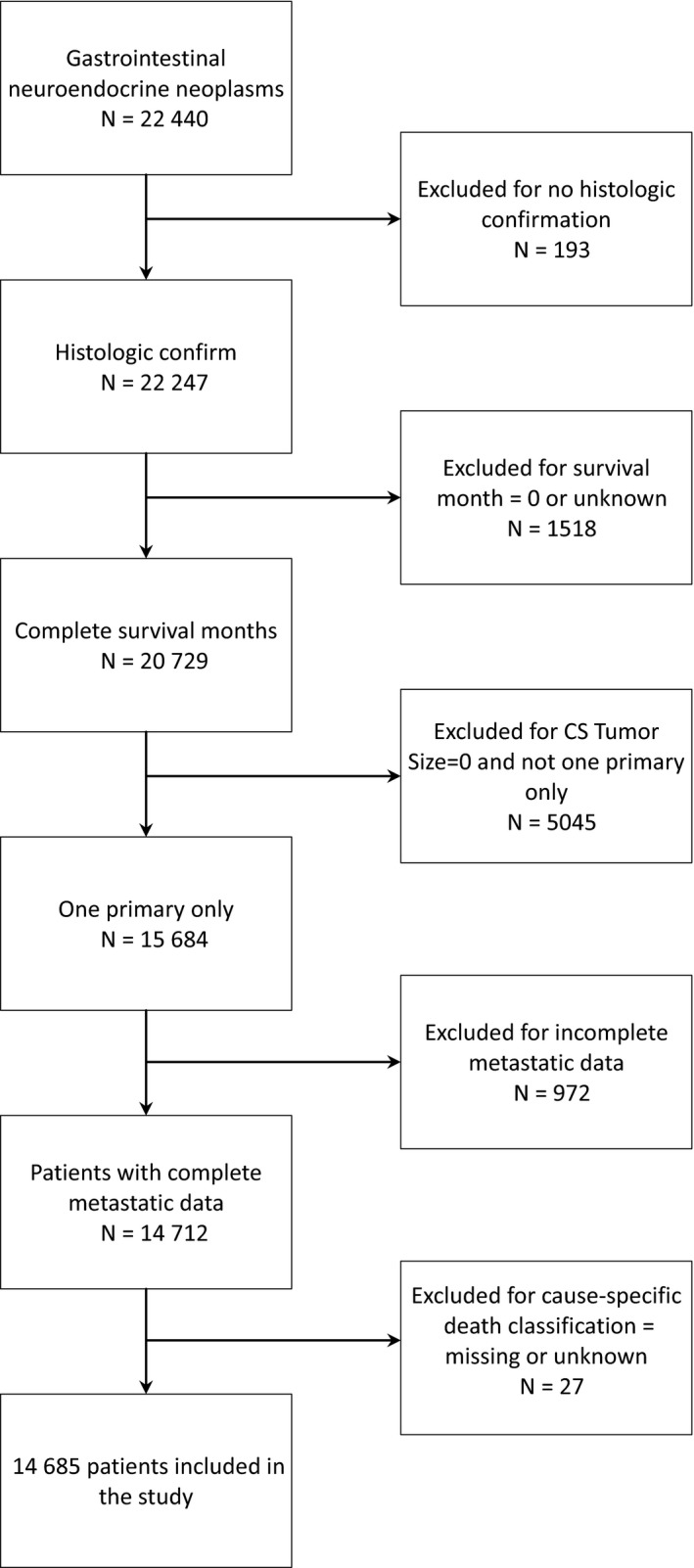
Flowchart of the inclusion and exclusion process. Total of 22 440 cases of gastrointestinal neoplasms (GI‐NENs) from SEER database were screened and 14 685 cases were included in the final analysis

Data from patients diagnosed with NEN of gastrointestinal tract from 1 January 2010 to 31 December 2015 were extracted from the SEER‐18 database with additional treatment fields. Using the International Classification of Diseases for Oncology (ICD‐O3) codes, the primary sites were defined including the esophagus, stomach, small intestine, appendix, colon, rectum, and anus. Histological types were defined by the following ICD‐O‐Histology/behavior codes: 8013/3, 8153/3, 8156/3, 8240/3, 8241/3, 8242/3, 8243/3, 8244/3, 8245/3, 8246/3, 8249/3, 8574/3 (variants of neuroendocrine tumors and carcinoids). The following SEER variables were collected: age, gender, year of diagnosis, ethnicity, marital status, insurance status, tumor grade and differentiation, tumor size, American Joint Committee on Cancer (AJCC) 7th TNM stage, surgery, radiotherapy, and overall survival. The GI‐NEN incidence data from the SEER‐9 registry for the years 1973 to 2015 were also obtained, and 22 440 GI‐NEN patients were initially identified. Of them, 193 patients were excluded because of incomplete pathological diagnosis. An additional 1545 patients were excluded due to missing survival details. In addition, multiple primary cancer patients and cases without records of metastatic data were further excluded (Figure 1). A final total of 14 685 patients were identified and included in the current study.

### 2.2 Statistical analysis

Joinpoint regression was performed using the Joinpoint software 4.5.0 (distributed by the Statistical Applications and Research Branch of the National Cancer Institute, Bethesda, MD; http://surveillance.cancer.gov/joinpoint). The percentage change (PC) and the annual percent change (APC) among different tumor locations were calculated from 1973 to 2015. Demographic, clinical information, and tumor features were summarized with descriptive statistics. Comparisons of categorical variables among different groups were performed using the chi‐squared test. The multivariate Cox proportional hazard model was used to assess the relative impacts of risk factors on metastasis in patients. Kaplan‐Meier survival curves were constructed for disease‐specific mortality and compared using the log‐rank test. All of statistical analyses were conducted via SPSS 25.0 (SPSS Inc), the Intercooled Stata SE 15.0 (Stata Corporation), and GraphPad Prism 7 (GraphPad Software). *P* < .05 was considered as statistically significant. APC was considered statistically significant when the regression line slope differed from zero at α = 0.50 level.

## 3 RESULTS

### 3.1 Incidence trend

**Figure 2:**
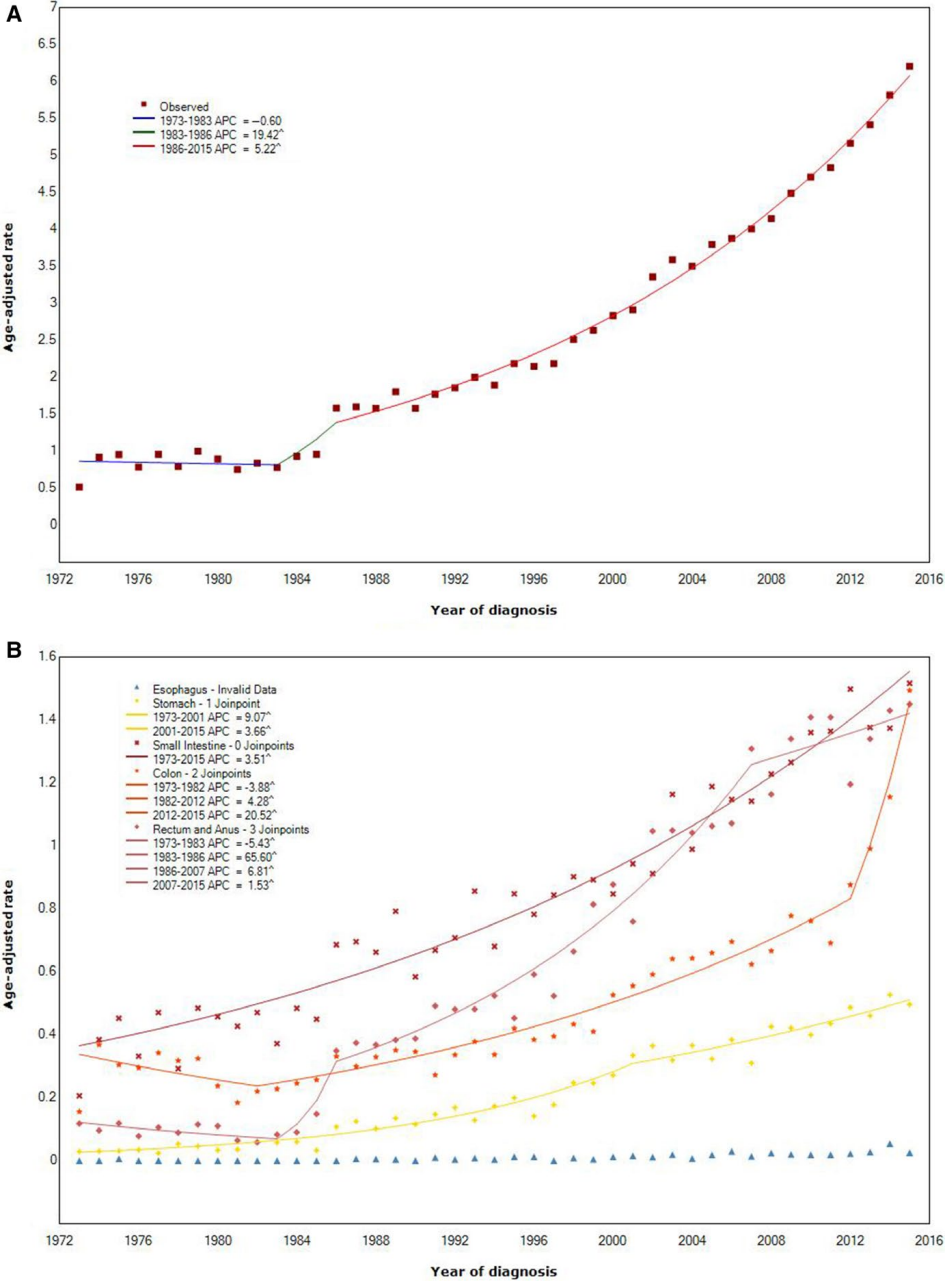
Incidence of gastrointestinal neuroendocrine neoplasms (GI‐NENs) from 1973 through 2015. Panel A: Age‐adjusted incidence of GI‐NENs. Panel B: Age‐adjusted incidence of NENs originated from various parts of gastrointestinal tract. (^) *P* < .05. APC: annual percentage change

GI‐NEN incidence increased from 0.51 per 100 000 patients in 1973 to 6.20 in 2015, with an APC of 19.42% (95% confidence interval (CI) = 7.9% to 32.1%, *P* < .001) from 1983 to 1986, and 5.22% from 1986 to 2015 (95% CI = 5.1% to 5.3%, *P* < .001), but there was no significant change in the incidence from 1973 to 1983 (Figure 2A).

Incidence of different locations of GI‐NENs was also assessed (Figure 2B). The incidence of gastric NENs increased significantly from 0.03 in 1973 to 0.50 in 2015, with an APC of 9.1% (95% CI = 8.7% to 9.5%, *P* < .001) from 1973 to 2001 and 3.7% from 2001 to 2015 (95% CI = 3.3% to 4.1%, *P* < .001). The incidence of intestinal NENs increased steadily from 0.21 in 1973 to 1.52 in 2015, with an APC of 3.5% (95% CI = 3.4% to 3.6%, *P* < .001). The incidence of colonic NENs decreased from 0.37 in 1974 to 0.22 in 1982 with a −3.9% APC (95% CI = −5.5% to −2.2%, *P* < .001) and increased from 0.22 in 1982 to 1.49 in 2015, with an APC at 4.3% (95% CI = 4.1% to 4.5%, *P* < .001) from 1982 to 2012 and 20.5% (95% CI = 17.0% to 24.1%, *P* < .001) from 2012 to 2015. For the rectal and anal NENs, the incidence also decreased from 0.12 in 1973 to 0.08 in 1983 with a −5.4% APC (95% CI = −7.8% to −3.0%, *P* < .001) and increased from 0.08 in 1983 to 1.45 in 2015, with an APC at 65.6% (95% CI = 27.6% to 114.9%, *P* < .001) from 1983 to 1986, 6.8% (95% CI = 6.5% to 7.1%, *P* < .001) from 1986 to 2007 and 1.5% (95% CI = 0.9% to 2.2%, *P* < .001) from 2007 to 2015.

### 3.2 Patient characteristics

The current study included 14 685 cases of GI‐NEN patients, including 7026 male patients (47.84%) and 7659 female patients (52.16%). The median age of the study group was 57 years old. The most frequent primary sites of NENs were the rectum (32.05%), small intestine (31.98%), and large intestine (24.30%). The incidence of stomach NEN (10.81%) was relatively lower, whereas the incidence of esophageal NENs (0.76%) was rare. According to American Joint Committee on Cancer (AJCC) 7th TNM stage classification, there were 123 (0.84%) patients at 0 stage, 5288 (36.01%) patients at stage I, 1751 (11.92%) patients at stage II, 2603 (17.73%) patients at stage III, 2003 (13.64%) patients at stage IV, and 19.86% patients were at unknown stage.

### 3.3 Metastasis of the tumors

Clinical feature of the GI‐NEN patients with liver, lung, bone and brain metastasis are presented in Table 1. Among the entire cohort, 1459 (9.94%) patients were diagnosed with liver metastasis, 144 (0.98%) patients with lung metastasis, 115 (0.78%) patients with bone metastasis, and 27 (0.18%) patients with brain metastasis. Patients who had metastasis to either one of the four sites constituted a high proportion of stage IV patients (74.64%).

**Table 1:** Clinical feature of the GI‐NEN patients with liver, lung, bone and brain metastasis

Features	Liver (%)			Lung (%)			Brain (%)			Bone (%)		
	0	1	*P*	0	1	*P*	0	1	*P*	0	1	*P*
Sex												
Male	6244 (88.9)	782 (11.1)	<.001	6939 (98.8)	87 (1.2)	.002	7009 (99.8)	17 (0.2)	.126	6953 (99.0)	73 (1.0)	.001
Female	6982 (91.2)	677 (8.8)		7602 (99.3)	57 (0.7)		7649 (99.9)	10 (0.1)		7617 (99.5)	42 (0.5)	
Age												
≤60	8069 (91.6)	742 (8.4)	<.001	8742 (99.2)	69 (0.8)	.003	8798 (99.9)	13 (0.1)	.240	8762 (99.4)	49 (0.6)	<.001
＞60	5157 (87.8)	717 (12.2)		5799 (98.7)	75 (1.3)		5860 (99.8)	14 (0.2)		5808 (98.9)	66 (1.1)	
Marital status												
Married	7097 (89.1)	869 (10.9)	.104	7894 (99.1)	72 (0.9)	.092	7945 (99.7)	21 (0.3)	.076	7898 (99.1)	68 (0.9)	.922
Unmarried	4682 (90.0)	522 (10.0)		5141 (98.8)	63 (1.2)		5198 (99.9)	6 (0.1)		5161 (99.2)	43 (0.8)	
Insurance												
Insured	12 006 (89.6)	1394 (10.4)	>.999	13 262 (99.0)	138 (1.0)	.803	13 375 (99.8)	25 (0.2)	.201	13 290 (99.2)	110 (0.8)	.781
Uninsured	381 (89.6)	44 (10.4)		422 (99.3)	3 (0.7)		423 (99.5)	2 (0.5)		421 (99.1)	4 (0.9)	
Race												
Caucasian	9243 (88.8)	1164 (11.2)	<.001	10 299 (99.0)	108 (1.0)	.327	10 385 (99.8)	22 (0.2)	.533	10 310 (99.1)	97 (0.9)	.010
African American	2447 (92.0)	213 (8.0)		2633 (99.0)	27 (1.0)		2658 (99.9)	2 (0.1)		2648 (99.5)	12 (0.5)	
Other	1099 (93.5)	77 (6.5)		1169 (99.4)	7 (0.6)		1174 (99.8)	2 (0.2)		1170 (99.5)	6 (0.5)	
Unspecific	437 (98.9)	5 (1.1)		440 (99.5)	2 (0.5)		441 (99.8)	1 (0.2)		442 (100)	0 (0.0)	
Primary tumor site												
Esophagus	67 (59.8)	45 (40.2)	<.001	99 (88.4)	13 (11.6)	<.001	107 (95.5)	5 (4.5)	<.001	98 (87.5)	14 (12.5)	<.001
Stomach	1451 (91.4)	136 (8.6)		1569 (98.9)	18 (1.1)		1582 (99.7)	5 (0.3)		1571 (99.0)	16 (1.0)	
Duodenum	1270 (95.8)	56 (4.2)		1320 (99.5)	6 (0.5)		1325 (99.9)	1 (0.1)		1323 (99.8)	3 (0.2)	
Jejunum and ileum	1727 (81.0)	406 (19.0)		2123 (99.5)	10 (0.5)		2133 (100)	0		2123 (99.5)	10 (0.5)	
Appendix	1982 (99.1)	18 (0.9)		1998 (99.9)	2 (0.1)		2000 (100)	0		1998 (99.9)	2 (0.1)	
Colon	587 (77.0)	175 (23.0)		727 (95.4)	35 (4.6)		755 (99.1)	7 (0.9)		745 (97.8)	17 (2.2)	
Rectum and anus	4554 (96.7)	153 (3.3)		4676 (99.3)	31 (0.7)		4705 (99.9)	2 (0.1)		4677 (99.4)	30 (0.6)	
Unspecific site	1588 (77.2)	470 (22.8)		2029 (98.6)	29 (1.4)		2051 (99.7)	7 (0.3)		2035 (98.9)	23 (1.1)	
Grade												
I	6889 (93.3)	498 (6.7)	<.001	7368 (99.7)	19 (0.3)	<.001	7387 (100)	0	<.001	7371 (99.8)	16 (0.2)	<.001
II	1441 (88.8)	182 (11.2)		1614 (99.4)	9 (0.6)		1621 (99.9)	2 (0.1)		1612 (99.3)	11 (0.7)	
III	527 (63.6)	301 (36.4)		770 (93.0)	58 (7.0)		815 (98.4)	13 (1.6)		787 (95.0)	41 (5.0)	
IV	171 (58.0)	124 (42.0)		273 (92.5)	22 (7.5)		293 (99.3)	2 (0.7)		279 (94.6)	16 (5.4)	
Unspecific	4198 (92.2)	354 (7.8)		4516 (99.2)	36 (0.8)		4542 (99.8)	10 (0.2)		4521 (99.3)	31 (0.7)	
Tumor size												
<1 cm	5598 (99.3)	42 (0.7)	<.001	5638 (99.9)	2 (0.1)	<.001	5640 (100)	0	<.001	5638 (99.9)	2 (0.1)	<.001
1‐2 cm	2242 (89.9)	251 (10.1)		2485 (99.7)	8 (0.3)		2492 (99.9)	1 (0.1)		2484 (99.6)	9 (0.4)	
2‐3 cm	1074 (81.4)	245 (18.6)		1310 (99.3)	9 (0.7)		1317 (99.8)	2 (0.2)		1310 (99.3)	9 (0.7)	
3‐4 cm	463 (73.8)	164 (26.3)		616 (98.2)	11 (1.8)		626 (99.8)	1 (0.2)		616 (98.2)	11 (1.8)	
4‐5 cm	256 (67.5)	123 (32.5)		365 (96.3)	14 (3.7)		377 (99.5)	2 (0.5)		370 (97.6)	9 (2.4)	
>5 cm	474 (64.9)	256 (35.1)		683 (93.6)	47 (6.4)		718 (98.4)	12 (1.6)		701 (96.0)	29 (4.0)	
Unspecific	3119 (89.2)	378 (10.8)		3444 (98.5)	53 (1.5)		3488 (99.7)	9 (0.3)		3451 (98.7)	46 (1.3)	
T‐stage												
Tis	123 (100)	0	<.001	123 (100)	0	<.001	123 (100)	0	.003	123 (100)	0	<.001
T1	5537 (99.4)	34 (0.6)		5566 (99.9)	5 (0.1)		5570 (99.9)	1 (0.1)		5565 (99.9)	6 (0.1)	
T2	1488 (89.8)	169 (10.2)		1645 (99.3)	12 (0.7)		1653 (99.8)	4 (0.2)		1646 (99.3)	11 (0.7)	
T3	2148 (81.7)	480 (18.3)		2602 (99.0)	26 (1.0)		2622 (99.8)	6 (0.2)		2601 (99.0)	27 (1.0)	
T4	1031 (73.3)	375 (26.7)		1371 (97.5)	35 (2.5)		1399 (99.5)	7 (0.5)		1383 (98.4)	23 (1.6)	
Unspecific	2899 (87.8)	401 (12.2)		3234 (98.0)	66 (2.0)		3291 (99.7)	9 (0.3)		3252 (98.5)	48 (1.5)	
N‐stage												
N0	9916 (96.3)	377 (3.7)	<.001	10 264 (99.7)	29 (0.3)	<.001	10 288 (99.9)	5 (0.1)	<.001	10 265 (99.7)	28 (0.3)	<.001
N1	2756 (76.0)	871 (24.0)		3547 (97.8)	80 (2.2)		3611 (99.6)	16 (0.4)		3565 (98.3)	62 (1.7)	
N2	113 (72.9)	42 (27.1)		151 (97.4)	4 (2.6)		153 (98.7)	2 (1.3)		150 (96.8)	5 (3.2)	
N3	9 (69.2)	4 (30.8)		11 (84.6)	2 (15.4)		12 (92.3)	1 (7.7)		11 (84.6)	2 (15.4)	
Unspecific	432 (72.4)	165 (27.6)		568 (95.1)	29 (4.9)		594 (99.5)	3 (0.5)		579 (97.0)	18 (3.0)	
Primary site surgery												
Yes	11 500 (93.1)	856 (6.9)	<.001	12 315 (99.7)	41 (0.3)	<.001	12 349 (99.9)	7 (0.1)	<.001	12 325 (99.7)	31 (0.3)	<.001
No	1621 (73.3)	590 (26.7)		2110 (95.4)	101 (4.6)		2191 (99.1)	20 (0.9)		2127 (96.2)	84 (3.8)	
Radiotherapy												
Yes	119 (75.3)	39 (24.7)	<.001	155 (98.1)	3 (1.9)	.202	150 (94.9)	8 (5.1)	<.001	151 (95.6)	7 (4.4)	<.001
No	13 107 (90.2)	1420 (9.8)		14 386 (99.0)	141 (1.0)		14 508 (99.9)	19 (0.1)		14 419 (99.3)	108 (0.7)	

The liver was the most frequent site of metastasis, comprising 72.84% of all M1 patients. Therein, solitary liver metastases accounted for 87.25% (1273/1459) of all liver metastases. Male patients and patients older than 60 years old had a significant higher percentage of liver metastasis. The marital status and insurance status were not significantly different between patients with or without liver metastasis. Among different ethnic groups, the percentage of patients with liver metastasis was highest in the Caucasian population (11.2%), while it was low in American Indian and Asian patients (6.5%). Esophageal NENs and colonic NENs had higher liver metastatic rates, while primary tumor at the stomach, duodenum, jejunum and ileum, appendix, and rectum showed less liver metastasis. As for tumor grading, the increase in liver metastatic rate was accompanied with higher tumor grading (lower level of differentiation). The increasing tumor size, T stage or N stage, was related to higher liver metastatic rates.

Characteristics for GI‐NEN patients with lung metastasis were similar to those with liver metastasis, which also has a modest gender bias favouring males and preference for older patients. There was no significant difference in the incidence of lung metastasis among patients of different ethnic backgrounds. Esophageal NENs had a higher lung metastatic rate than NENs of other origin.

Analysis on the incidence of brain metastasis data revealed that there was no significant difference among patients of different gender, age (>60 and younger), race, marital status, and insurance status. Esophageal NEN patients had the highest rate of brain metastasis (12.5%), while appendiceal NENs had the lowest rate (0.1%). Interestingly, there was a trend toward increasing brain metastatic rate as the tumor grade increases from grade I to III, but it showed a decrease in grade IV NENs.

For the patients with bone metastasis, male patients had significantly higher rate of incidence compared with the female patients. Median age of the patients who had bone metastasis was 5 years older than those without. For ethnicity, Caucasian patients had a higher percentage of bone metastasis than other ethnic patients. Esophageal NEN patients had the highest bone metastatic rate (4.5%), while there were no reported cases of bone metastasis in appendiceal, jejunal, and ileal NENs. For the advanced T and N stage patients, the bone metastatic rate was higher.

Among the 2003 patients who developed metastasis of the tumor, 1305 had a specific record of single metastasis to the liver, lung, bone or brain, 508 patients staged M1 without a description of the metastatic site and 190 patients developed multiple organ metastases. All of the various combinations of these four sites of metastases are shown in Table 2. The most common site of concurrent metastases was the liver and lung (0.6%). Only six (0.04%) patients experienced metastases to all four sites at the same time.

**Table 2:** Summary of metastasis sites in liver, lung, bone, and brain

	Gastrointestinal tract (%) N = 14 685	Esophagus (%) N = 112	Stomach (%) N = 1587	Duodenum (%) N = 1326	Jejunum and ileum (%) N = 2133	Appendix (%) N = 2000	Colon (%) N = 762	Rectum and anus (%) N = 4707	Unspecific site (%) N = 2058
No metastasis	12 624 (86.0)	54 (48.2)	1419 (89.4)	1252 (94.4)	1559 (73.1)	1877 (93.9)	539 (70.7)	4500 (95.6)	1425 (69.2)
One site									
Only liver	1253 (8.5)	29 (25.9)	109 (6.9)	49 (3.7)	394 (18.5)	16 (0.8)	138 (18.1)	98 (2.1)	420 (20.4)
Only lung	28 (0.2)	2 (1.8)	3 (0.2)	2 (0.2)	3 (0.1)	2 (0.1)	8 (1.0)	4 (0.1)	4 (0.2)
Only bone	20 (0.1)	6 (5.3)	2 (0.1)	1 (0.1)	4 (0.2)	0	2 (0.3)	1 (0)	4 (0.2)
Only brain	4 (0)	0	0	0	0	0	2 (0.3)	0	2 (0.1)
Two sites									
Liver and lung	86 (0.6)	6 (5.4)	10 (0.6)	4 (0.3)	6 (0.3)	0	21 (2.8)	19 (0.4)	20 (1.0)
Liver and bone	62 (0.4)	3 (2.7)	10 (0.6)	2 (0.2)	5 (0.2)	2 (0.1)	9 (1.2)	18 (0.4)	13 (0.6)
Liver and brain	9 (0.1)	3 (2.7)	2 (0.1)	1 (0.1)	0	0	1 (0.1)	0	2 (0.1)
Lung and bone	3 (0)	1 (0.9)	0	0	1 (0)	0	0	1 (0)	0
Lung and brain	1 (0)	0	0	0	0	0	1 (0.1)	0	0
Bone and brain	2 (0)	0	0	0	0	0	0	1 (0)	1 (0)
Three sites									
Liver and lung and bone	16 (0.1)	2 (1.8)	2 (0.1)	0	0	0	3 (0.4)	6 (0.1)	3 (0.1)
Liver and lung and brain	1 (0)	0	1 (0.1)	0	0	0	0	0	0
Liver and bone and brain	3 (0)	0	0	0	0	0	1 (0.1)	1 (0)	1 (0)
Lung and bone and brain	1 (0)	0	0	0	0	0	1 (0.1)	0	0
Four sites	6 (0)	2 (1.8)	2 (0.1)	0	0	0	1 (0.1)	0	1(0)
Metastasis to other sites	508 (3.5)	5 (4.5)	27 (1.7)	15 (1.1)	160 (7.5)	103 (5.2)	35 (4.6)	15 (0.3)	148 (7.2)

Furthermore, we compared the occurence of concurrent metastases at the time of diagnosis among different locations. Patients with liver metastasis were more likely to have metastasis at bone (6.10% vs 0.20%, odds ratio [OR] = 32.98, 95% CI = 21.42 to 50.78, *P* < .001) or brain (1.30% vs 0.06%, OR = 21.8, 95% CI = 9.85 to 47.18, *P* < .001) than patients without liver metastasis. Also, patients with lung metastasis also had a higher risk of metastating to bone (18.06% vs 0.61%, OR = 35.78, 95% CI = 22.29 to 56.4, *P* < .001) or brain (6.25% vs 0.12%, OR = 53.79, 95% CI = 23.74 to 120, *P* < .001) than the patients without lung metastasis. Unfortunately, however, the chronological order of metastasis was not recorded in the database.

### 3.4 Survival

**Figure 3:**
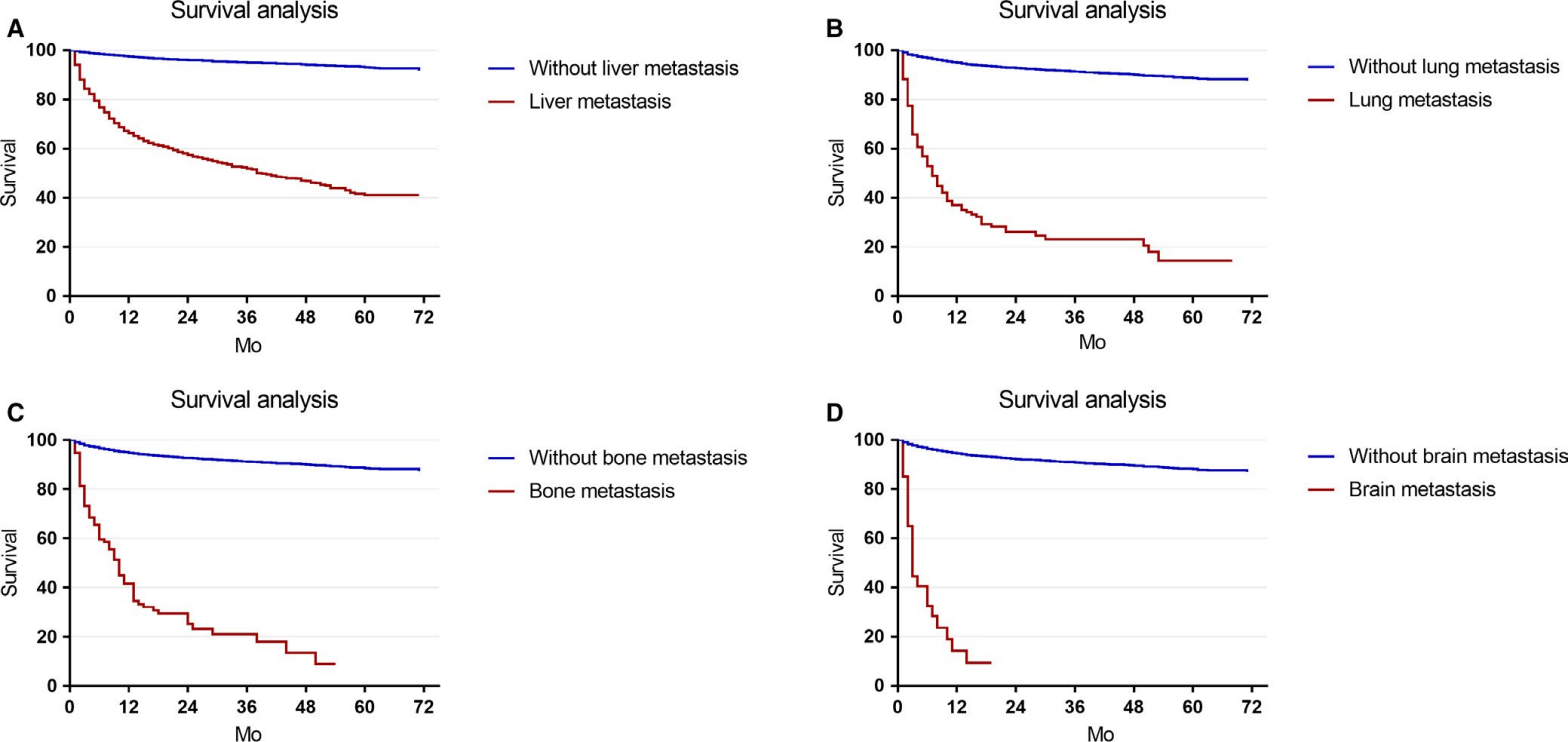
Survival analyses of the GI‐NEN patients with liver, lung, bone or brain metastasis. Panel A: Patients with or without liver metastasis, *P* < .001. Panel B: Patients with or without lung metastasis, *P* < .001. Panel C: Patients with or without bone metastasis, *P* < .001. Panel D: Patients with or without brain metastasis, *P* < .001

In this study, 1184 cancer‐specific deaths (8.06%) were observed. The 5‐year cause‐specific survival (CSS) was 87.95% in the study cohort. The 5‐year CSS was 41.14% vs 93.19% for patients with or without liver metastasis (*P* < .001, Figure 3A), and 14.44% vs 88.68% for patients with or without lung metastasis (*P* < .001, Figure 3B). The 3‐year CSS was 20.99% vs 91.32% for patients with or without bone metastasis (*P* < .001, Figure 3C). And the 1‐year CSS was 14.20% vs 94.74% for patients with or without brain metastasis (*P* < .001, Figure 3D). The median overall survival (OS) time for patients with liver, lung, bone, and brain metastasis was 38, 6, 9, and 2 months, respectively.

### 3.5 Risk factors for GI‐NENs patients with liver metastasis

**Figure 4:**
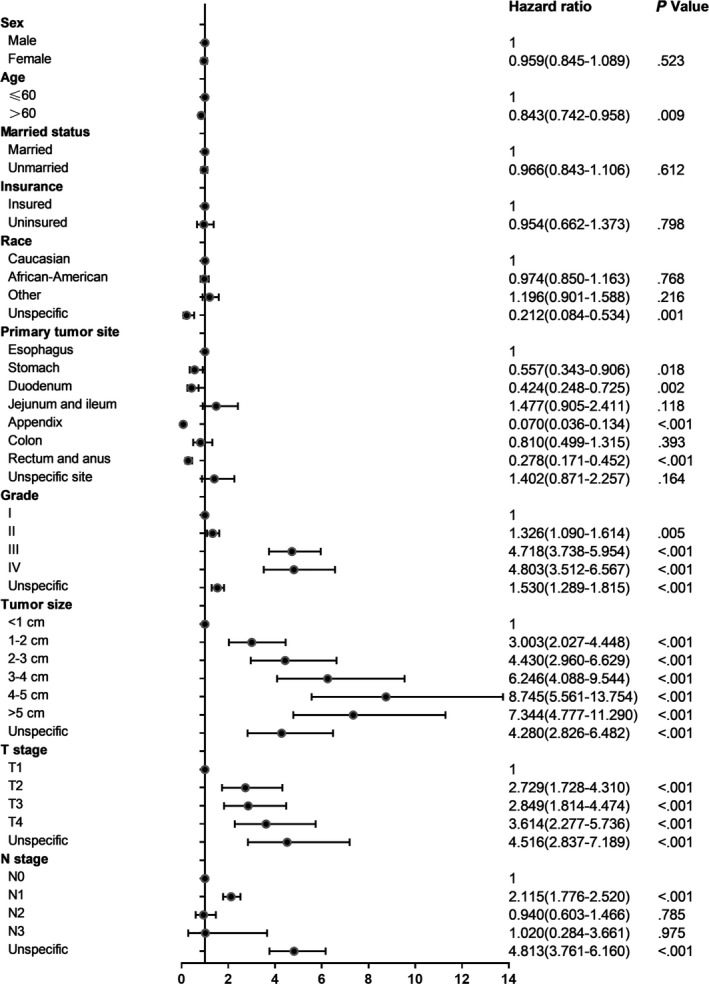
Forest plot of multivariate regression analysis for liver metastasis of GI‐NENs. Horizontal axis: Hazard ratio on a log scale with the reference line, Hazard ratios (circle) and 95% CI (whiskers)

In accordance with earlier studies, the liver was the most common site of GI‐NEN metastasis. Thus we performed multivariate regression to further explore the risk factors in developing liver metastasis from GI‐NENs and summarized the results in the forest plot (Figure 4). It was found that increased tumor grading, larger tumor size, advanced T stage and N1 stage could significantly increase the risk of liver metastasis. In addition, esophageal, jejunal, ileac, and colonic NENs had higher risk of liver metastasis.

## 4 DISCUSSION

In this study, pattern of the GI‐NEN metastasis and the risk factors for liver metastasis were analyzed. A significant increase in age‐adjusted incidence of GI‐NENs from 0.51 per 100 000 in 1973 to 6.20 per 100 000 in 2015 was observed. Majority of the patients were at an advanced stage at the time of diagnosis. Thus, a better understanding of NEN diagnosis and its metastatic pattern becomes even more important for clinicians in order to make better decision at pretreatment evaluation stage.

The current study found that metastatic rate of GI‐NENs to liver, lung, bone and brain at the time of diagnosis was 9.94%, 0.98%, 0.78%, and 0.18%, respectively, and that patients with lung or liver metastasis had a higher risk of co‐existing bone and brain metastasis at the same time than those without lung or liver metastasis. These findings suggested that metastasis of GI‐NEN to bone or brain be alerted if a GI‐NEN patient had liver or lung metastasis. Findings of the current study on the metastatic patterns of GI‐NENs could provide better guidance for the clinicians to develop an evaluation strategy of GI‐NENs at the time of diagnosis. For instance, considering cost‐effects, the bone and brain might not be screened at the time of GI‐NEN diagnosis; when the patient had liver and lung metastasis; however, bone and brain should be examined to exclude GI‐NEN metastasis.

The current study has also explored the risk factors of GI‐NEN metastasis. NENs originating from the esophagus had the highest rate of metastasis compared to the NENs from other parts of gastrointestinal tract. For the liver metastasis of GI‐NENs, the patients with lower differentiation of GI‐NENs had higher rate of liver metastasis. For the lung, bone, and brain metastasis, however, significant increase in metastatic rate only occurred in poorly differentiated or undifferentiated NENs (Grade III and Grade IV), while well or moderately differentiated NENs (Grade I and Grade II) had a much smaller metastatic rate. Caucasian patients had the highest risk of liver and bone metastasis, and American Indian and Asian patients had the lowest possibility to develop any metastasis. Increasing tumor size, T stage and N stage, was related to the higher risk of NEN metastasis. Insurance and marital status had no influence on the metastasis of GI‐NENs to the liver, lung, bone and brain in the current study, although earlier reports showed that insured patients might receive more early examination and married patients tend to undergo earlier testing and receive more adequate treatment.^9^, ^10^, ^11^, ^12^, ^13^, ^14^

Using the SEER database from 1973 through 2012, Dasari and colleagues has analyzed incidence trends and survival rate.^2^ They found that the incidence and prevalences of neuroendocrine tumors (NET) were steadily rising and the survival for all NETs has improved over time, especially for distant‐stage gastrointestinal NETs and pancreatic NETs in particular.^2^ However, SEER data of GI‐NEN metastasis to the liver, bone, brain and lung were not available until 2010. Here, we further extracted data from SEER‐18 database from 1 January 2010 to 31 December 2015 for the patients diagnosed with GI‐NEN, and particularly analyzed data of metastasis to liver, lung, bone, and brain. We found that incidence of GI‐NENs increased from 0.51 per 100 000 patients in 1973 to 6.20 per 100 000 patients in 2015, and that 72.84%(1459/2003) patients had liver metastasis, 7.19% (144/2003) had lung metastasis, 5.74% (115/2003) had bone metastasis, and 1.35% (27/2003) had brain metastasis. The rising of incidence of GI‐NENs and its metastisis could be associated with newly developed imaging systems such as Ga^68^ DOTATATE PET, which appears to be more sensitive in detecting distant metastasis.^15^, ^16^, ^17^

To the best of our knowledge, this is the first SEER‐based study focusing on the metastatic patterns, risk factors of metastasis in GI‐NEN patients with the longest follow‐up time. However, there were some limitations. First of all, chemotherapy information, which was an important treatment related to survival, was not available in the database. Second, we only had the metastasis information at the time of diagnosis. Some patients might have developed metachronous metastatic lesions, which might lead to an undervalue of metastatic status. Third, distant metastasis data were not available earlier than 2010 in SEER database, which limited the analysis on the outcomes of GI‐NEN patients survival and prognosis after the treatment. Fourth, impact of newer agents in overall treatment was unable to analyze in this study because we could not obtain the data of targeted chemotherapy from the database. Finally, we could not analyze the impact of surgery, chemotherapy, or radiotherapy on the survival of the NENs because the number of patients who received radiotherapy or chemotherapy with or without surgery was unclear in the database (category of “unknown” patients in the database).

## 5 CONCLUSIONS

This population‐based study, which was based on the SEER database, provided evidence of increasing incidence of GI‐NENs and identified the risk factors of GI‐NEN metastasis to the liver, lung, bone, or brain. While liver was the most common site of GI‐NEN metastasis, patients with liver metastasis had relatively better prognosis. In contrast, patients with brain, bone, and lung metastasis of GI‐NENs had poor prognosis. GI‐NEN patients were very likely to have bone and brain metastasis if they had lung or liver metastasis. Findings of the current study could help clinicians to identify distant metastasis of GI‐NENs as early as possible, and by which, to improve the survival rate of GI‐NENs.

## CONFLICT OF INTEREST

The authors declared no conflict of interest.
